# Formation of the Ascidian Epidermal Sensory Neurons: Insights into the Origin of the Chordate Peripheral Nervous System

**DOI:** 10.1371/journal.pbio.0040225

**Published:** 2006-06-27

**Authors:** Andrea Pasini, Aldine Amiel, Ute Rothbächer, Agnès Roure, Patrick Lemaire, Sébastien Darras

**Affiliations:** **1**Institut de Biologie du Développement de Marseille-Luminy (IBDML), UMR6216, CNRS/Université de la Méditerranée, Marseille, France; University of California BerkeleyUnited States of America

## Abstract

The vertebrate peripheral nervous system (PNS) originates from neural crest and placodes. While its developmental origin is the object of intense studies, little is known concerning its evolutionary history. To address this question, we analyzed the formation of the larval tail PNS in the ascidian Ciona intestinalis. The tail PNS of *Ciona* is made of sensory neurons located within the epidermis midlines and extending processes in the overlying tunic median fin. We show that each midline corresponds to a single longitudinal row of epidermal cells and neurons sharing common progenitors. This simple organization is observed throughout the tail epidermis, which is made of only eight single-cell rows, each expressing a specific genetic program. We next demonstrate that the epidermal neurons are specified in two consecutive steps. During cleavage and gastrula stages, the dorsal and ventral midlines are independently induced by FGF9/16/20 and the BMP ligand ADMP, respectively. Subsequently, Delta/Notch–mediated lateral inhibition controls the number of neurons formed within these neurogenic regions. These results provide a comprehensive overview of PNS formation in ascidian and uncover surprising similarities between the fate maps and embryological mechanisms underlying formation of ascidian neurogenic epidermis midlines and the vertebrate median fin.

## Abstract 2

In this comprehensive study of sensory neuron development in the ascidian tadpole, FGF, BMP, and Notch signals act to specify peripheral sensory neurons by a two-step inductive process along the dorsal and ventral midlines.

## Introduction

The peripheral nervous system (PNS) allows animals to receive a large part of their information from the environment and is thus essential to adjust their behavior to external cues. The developmental origin of the PNS has been the object of intensive studies in both vertebrates and invertebrates. In vertebrates, most sensory neurons of the PNS originate from the neural crest and neurogenic placodes, two specialized ectodermal derivatives that lie at the border of the neural plate [ 1]. Neural crest and placodes arise through multiple developmental steps, controlled by complex gene regulatory networks [ 2– 8]. First, the neural plate border is specified by signals from the surrounding ectoderm (neural plate and epidermis) and from the underlying endomesoderm. These signals include bone morphogenetic protein (BMP), fibroblast growth factor (FGF), Wnt, and Notch. Within the neural plate border, the action of specifier genes controls the acquisition of placodal- and neural crest–specific phenotypes, notably the ability to undergo cell shape changes leading to placodal cell delamination and long-range neural crest cell migration. Finally, the diversity of the placodal and neural crest derivatives is generated by the combinatorial action of extrinsic and intrinsic cues.

Neural crest and placodes are commonly thought to be vertebrate specific innovations having a key evolutionary role. According to the so-called New Head Hypothesis, the acquisition of novel placode- and neural crest–derived structures could have been instrumental in the shift from filter feeding to the active predatory lifestyle typical of vertebrates [ 9]. The protochordates (cephalochordates and urochordates) are a chordate group basal to vertebrates, with urochordates being the closest relatives of vertebrates, according to recent phylogenetic analyses [ 10, 11]. Thanks to their phylogenetic position, these organisms may help us to understand the evolutionary origin of the vertebrate PNS. Although there are some reports that ascidians (urochordates) may have both neural crest–like cells [ 12] and placode-like structures [ 13, 14], these studies are mainly based on gene expression profiles and morphological data, with little or no functional analysis, and their conclusions are controversial [ 9, 15]. It is generally thought that placodes and neural crest did not appear de novo in vertebrates, but originate from cell populations already present in protochordates. The key neural crest or placodes features such as migratory ability or pluripotency, absent in protochordates, would have been acquired later, possibly through gene cooption [ 16– 21]. The ascidian dorsal midline tail epidermis has been proposed to be an evolutionary precursor of the neural crest, since it gives rise to sensory neurons and expresses homologues of neural plate border markers but not homologues of either neural crest or placodes specifier genes [ 3, 13, 18– 21].

To get better insights into plausible scenarios of vertebrate PNS evolution, we chose to study the developmental mechanisms controlling the formation of larval PNS in the ascidian Ciona intestinalis. Ascidian larvae combine the key chordate features of a notochord flanked by muscle bands and a dorsal hollow neural tube with a very simple body plan: the number of tissues and organs is limited, and the total cell number is generally fewer than 3,000.

The PNS of *Ciona* larvae is composed of a limited number of epidermal sensory neurons (ESN) located in the trunk and tail [ 22]. The tail PNS is made of ESNs (here referred to as caudal ESNs or CESNs) scattered at more or less regular intervals along the ventral and dorsal midlines. All CESNs extend long processes in the cellulose-based fin tunic that envelopes the larva and are likely to be mechanosensors controlling swimming behavior [ 23, 24]. These cells have been described in several ascidian species [ 22, 23, 25], but their developmental history and specification mechanisms are still poorly understood. It has been shown that ESNs are induced by signals from the vegetal hemisphere which can be mimicked by bFGF [ 25, 26]. Consistent with a possible role for FGF signalling, extracellular signal-regulated kinase (Erk) activity is required for proper formation of the PNS [ 27]. In addition, activation of the Notch pathway leads to a loss of ESNs [ 28].

Here, we present the first comprehensive description of the cellular and molecular events required for the formation of the ascidian PNS, from induction to terminal differentiation. We found that CESN generation can only be understood as part of an overall process of medio-lateral patterning of the tail epidermis and that it requires at least two developmental events: 1) dorsal and ventral midline regions are specified by *fgf9/16/20*- *nodal* and *admp* signals, respectively; 2) within these neurogenic regions, the fate of the CESN precursors is controlled by the Delta/Notch pathway. Finally, our results point to an unexpected connection between PNS development and lower vertebrate median fin formation.

## Results

### Organization and Morphology of Tail Epidermal Neurons

**Figure 1:**
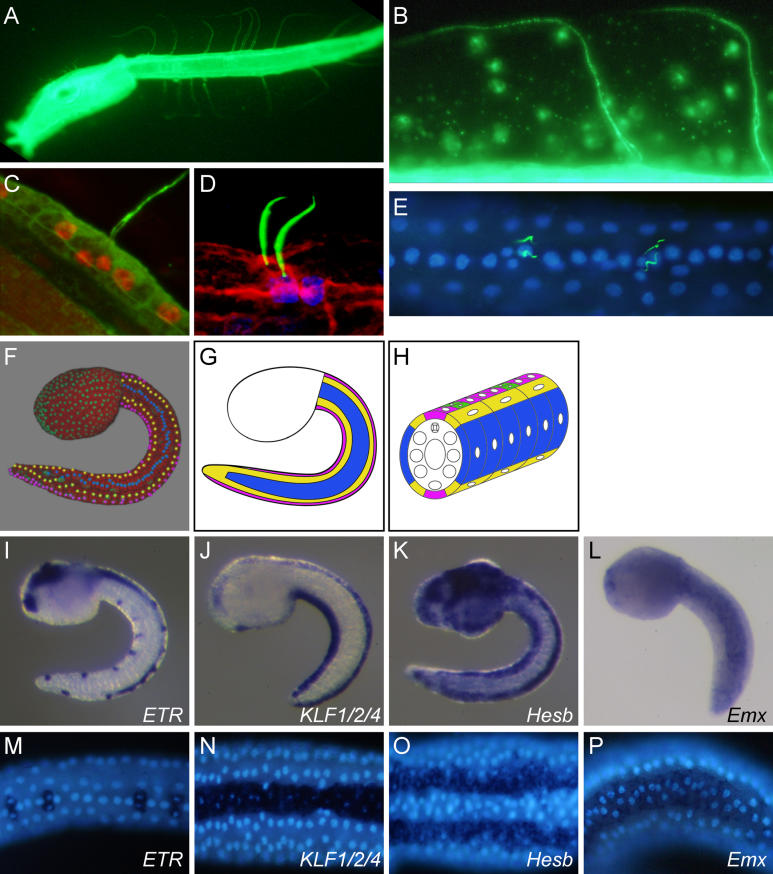
CESNs and Tail Epidermis Medio-Lateral Patterning (A–C) β-tubulin immunostaining showing CESN projections in the fin tunic. Confocal images projection with nuclei in red (propidium iodide) (C). (D and E) Acetylated α-tubulin showing the proximal part of the projections from paired CESNs (Acetylated α-tubulin in green, DAPI in blue, phalloidin in red). (F) 3-D projection extracted from a time lapse movie of an embryo injected with H2B-YFP mRNA and Fast Green (red). Trunk nuclei are green, while tail nuclei are colored to highlight the longitudinal rows made of single cell. (G and H) Schematic representation of the tail medio-lateral patterning (medial row, purple; medio-lateral row, yellow; lateral row, blue). (I–P) Expression patterns of tail epidermis markers at the mid-tailbud stage. Lateral view (I–L and P). Ventral view (M–O). Anterior is to the left. DAPI staining of nuclei in (M–P).

The caudal epidermal sensory neurons (CESNs) constituting the tail PNS are easily identified by their long, β-tubulin-positive projections extending into the dorsal and ventral fin tunic [ 24] ( Figure 1A– 1C). Distally, these extensions bend to follow the fin outline and are exposed to the seawater ( Figure 1B). Their proximal portion is specifically stained by acetylated α-tubulin immunodetection ( Figure 1D and 1E). The CESN cell body is embedded in the epidermis and presents a narrow apical surface from which the projection arises ( Figure 1C and 1D). Remarkably, the CESNs are always found as pairs ( Figure 1D and 1E), except for a single CESN at the very posterior ventral tip of the tail. The two nuclei of a CESN pair are smaller than the surrounding epidermal nuclei and lie on a line which always deviates from the antero-posterior axis of the embryo. Therefore, CESNs can be easily identified by simple nuclear staining. The number of CESN pairs varies among individual larvae (eight to 20 pairs with an average of 14 pairs in total, *n* = 74 larvae analyzed). Pairs of CESNs are scattered at more or less regular intervals along the midlines, intercalated by three to five rectangular epidermal cells with a large nucleus ( Figure 1E). Thus, ventral and dorsal midlines are made of single rows of epidermal cells with interspersed pairs of CESNs.

### Medio-Lateral Pattern of the Tail Epidermis: Cellular Arrangement and Molecules

In the tail epidermis, the stereotyped cell organization is not limited to the midlines. Visualization of nuclei, in fixed ( Figure 1E) or live specimens ( Figure 1F), reveals a strikingly regular architecture at tailbud and hatching stages. Tail epidermis cells are organized according to three different tiling patterns ( Figure 1F– 1H). The dorsal and ventral midlines are each constituted of a single row of cells elongated along the antero-posterior axis. They are flanked bilaterally by medio-lateral domains, each constituted of a single row of flat cells with large nuclei. Each flank of the tail is made up of a single row of long and flat rectangular cells, stretched along the dorso–ventral axis of the tail and constituting a lateral domain. Thus, eight distinct single-cell rows (two medial domains, four medio-lateral domains, and two lateral domains) make up most of the *Ciona* larval tail epidermis. This regular organization is partially lost around the tapering tip of the tail.

Search through available expression pattern databases and published literature reveals that many C. intestinalis genes expressed in the tail epidermis show regionalized patterns of expression (Ghost database: http://ghost.zool.kyoto-u.ac.jp; Aniseed database: http://aniseed-ibdm.univ-mrs.fr) [ 29, 30]. To check whether this molecular regionalization corresponds to the observed morphological regionalization of the tail epidermis, we performed whole-mount ISHs followed by DAPI staining on tailbud stage embryos. We identified several genes whose expression within the tail epidermis is restricted to the medial, the medio-lateral, or the lateral domain. Figure 1I– 1P shows representative examples. The Krüppel-like zinc finger transcription factor *KLF1/2/4* (referred to as *zinc finger transcription factor (C2H2)-24* in [ 30], but phylogenetic analysis show affinities with the group formed by human *KLF1, KLF2,* and *KLF4* [ 31]) is specifically expressed in both the dorsal and ventral midlines. The hairy-related bHLH transcription factor *Hesb* is expressed in the dorsal and ventral medio-lateral domains. The homeobox-containing transcription factor *emx* is bilaterally expressed in the lateral domains. Other genes are specifically expressed in the CESNs themselves ( *β-thymosin-like, ETR,* or *POU-IV* [ 32], Figure 1I and 1M). The list of genes we identified with restricted pattern can be found in Table S1. Thus, each of the three morphological domains of the *Ciona* larval tail epidermis appears to be defined by a specific genetic program.

### A Clonal Basis for Tail Epidermis Regionalization in *Ciona*

**Figure 2:**
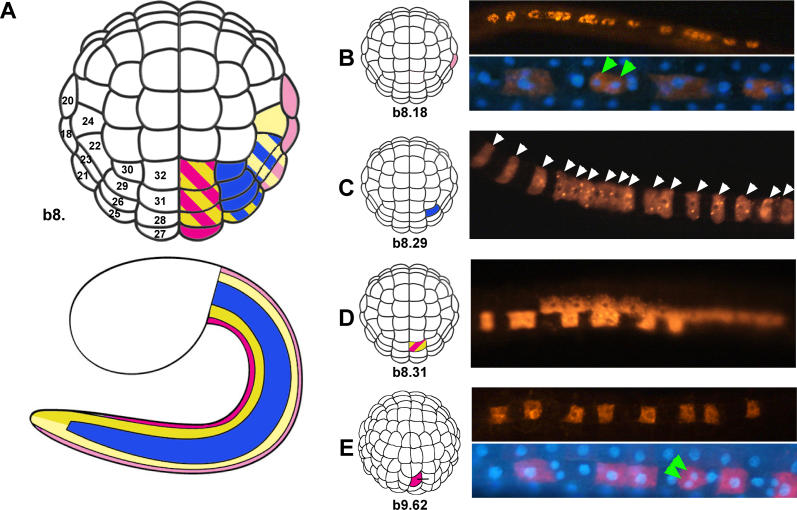
Cell Lineage of the Tail Epidermis in *Ciona* (A) Color-coding illustrates the clonal base of dorso–ventral regionalization of tail epidermis. Pink, dorsal midline; light yellow, dorso–lateral domain; blue, lateral domain; yellow, ventro-lateral domain; purple, ventral midline. For a detailed description of each 110-cell stage b–line blastomere's contribution to the tail epidermis, see Figure S1. (B–E) composition of four representative clones derived from DiI-labelled epidermal precursors. (C) a lateral domain clone consists of 16 epidermal cells. (B and E) Dorsal (B) and ventral (E) midine clones consist of both epidermal cells and CESN pairs (green arrowheads). See Table S2 for a quantification of midline clone composition. (B) The progeny of a 110-cell stage dorsal precursor only contributes to the medial domain. (D) The progeny of a 110-cell stage ventral precursor contributes to both medial and medio-lateral domains. (E) A single-cell division segregates the ventral medial and medio-lateral domains.

As a first step towards understanding tail epidermis regionalization in *Ciona,* we undertook a cell lineage analysis. Lineage-tracing experiments in Halocynthia roretzi have previously shown that the tail epidermis is derived from the posterior animal hemisphere b-line cells at the 110-cell stage (beginning of gastrulation) [ 33]. We individually labelled with DiI all the *Ciona* b-line blastomeres at the 110-cell stage, with the exception of the two blastomeres, b8.19 and b8.17, for which a contribution to the epidermis has already been ruled out [ 33, 34], and analyzed the size and distribution of their progeny at the late tailbud/hatching larva stage. The results, summarized in Figure 2, Figure S1, and Table S2, reveal a clonal basis for the medio-lateral organization of *Ciona* tail epidermis.

While the clones that contribute to the medial domains are composed of epidermal cells and CESNs in variable numbers (see next section), all other clones invariably contain 16 epidermal cells ( Figure 2C and Table S2). This is consistent with each precursor undergoing four rounds of cell division between the 110-cell stage and the late tailbud/hatching larva stage, as previously inferred for H. roretzi [ 33]. Of the 14 labelled b-line blastomeres, seven are already fate restricted at the 110-cell stage, while the remaining seven participate in only two different types of domain. In most cases (6/7), the progeny of these non-restricted blastomeres is equally distributed between the two domains (eight cells/domain), suggesting that one cell division is sufficient for these domains to segregate. This could be confirmed for the two blastomeres b8.31 and b8.28. As shown in Figure 2E and Table S2, their cleavage is perpendicular to the antero-posterior axis of the embryo and segregates the ventral medial and ventral medio-lateral domains.

During the processes of gastrulation, neurulation, and tail elongation, the precursors of the various domains of the tail epidermis undergo morphogenetic movements to end up as single-cell rows. In particular, the rolling up of the posterior neural tube brings about the juxtaposition of the left and right precursors of dorsal medial domain which intercalate extensively ( Figure 2B). Intercalation also takes place between the left and right precursors of the ventral medial domain ( Figure 2D and 2E).

### Each Midline Precursor Can Generate Both Epidermal Cells and CESNs

As presented above, the blastomeres contributing to the dorsal or ventral medial domains give rise to mixed populations of CESNs and epidermal cells ( Figure 2B and 2E and Table S2). Interestingly, the two cells within each CESN pair were always labelled together, suggesting that they are sister cells. We were able to track the formation of CESN pairs by time-lapse confocal imaging with a nuclear form of YFP. From the mid-tailbud stage onwards, no cell division occurs in the tail epidermis, except at scattered positions in the ventral and dorsal midlines where they take place along a plane perpendicular to the antero-posterior axis (unpublished data). The neurons in each pair are thus sister cells that arise from a late division. The number of individual cells within each midline clone varies from blastomere to blastomere as well as when the same blastomere is labelled in different embryos ( Table S2). However, if we take into account the fact that paired neurons are sister cells, the number of cells within each clone before the last division becomes invariant. In most cases, like for the other epidermis lineages, this number is 16.

These data can be most easily explained assuming that each 110-cell stage epidermis precursor generates a clone of invariant size by the mid-tailbud stage; subsequently, a variable number of cells within each midline clone undergo a supplementary fifth division to give rise to pairs of CESNs.

### FGF or BMP Pathway Activation Induces Ectopic Midline Identity and ESN Formation

**Figure 3:**
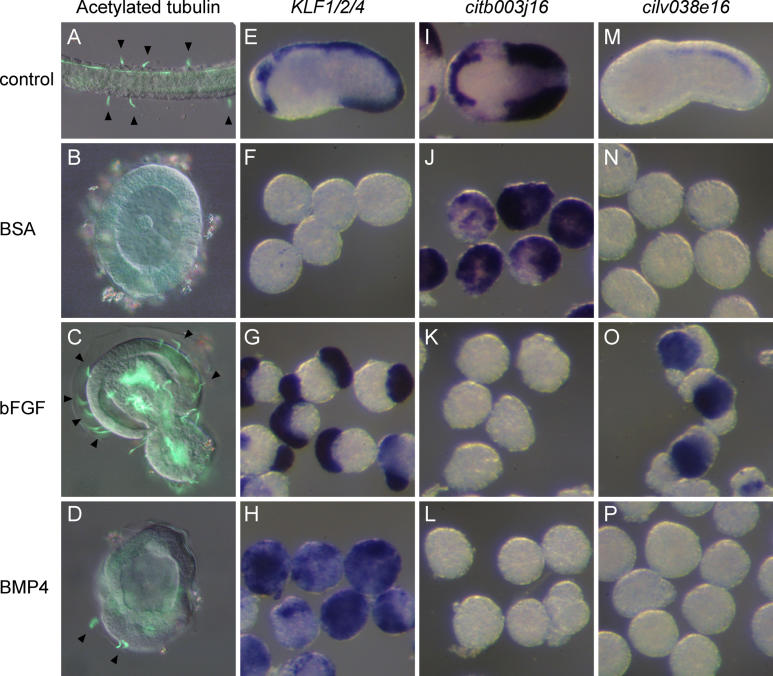
bFGF and BMP4 Induce Midline Fate and ESN Formation in Isolated b-Line Explants b4.2 Blastomeres were isolated at the eight-cell stage and treated with proteins from the 16-cell stage. (A–D) Acetylated α-tubulin immunostaining at larval stage: CNS and PNS structures are labelled. Arrowheads indicate ESNs. (E–P) Molecular marker expression at early tailbud stages: midline marker *KLF1/2/4* (E–H), lateral and medio-lateral marker *citb003j16* (I–L), and tail nerve cord marker *cilv038e16* (M–P). Top row, whole embryos (A,E,M) lateral view, (I) dorsal view, anterior to the left. Second row, control b4.2 blastomeres treated with BSA. Third row, b4.2 blastomeres treated with bFGF protein. Bottom row, b4.2 blastomeres treated with BMP4 protein.

In both *Ciona* and *Halocynthia,* formation of ESNs requires inductive interactions from the A- and B-line vegetal cells [ 25, 26]. We readdressed this issue with the help of our various markers ( Figure 3). When the precursors of the tail epidermis (b4.2 blastomeres) were isolated at the eight-cell stage, they developed into tunic-producing, acetylated α-tubulin-negative epidermis at larval stages ( *n* = 38; Figure 3B). Interestingly, these explants did not express the midline marker *KLF1/2/4* (only 5% of weakly positive explants, *n* = 21; Figure 3F) nor the medio-lateral marker *Hesb* (17% of weakly positive explants, *n* = 18; unpublished data) at the early tailbud stage. Instead, they adopted a lateral identity as revealed by the expression of *citb003j16,* a marker of the lateral and medio-lateral domains [ 29] (89% of positive explants, *n* = 27; Figure 3I and 3J). Thus, the entire midline fate, and not only the CESNs, depends on signals from the vegetal hemisphere.

We tested whether two signalling pathways involved in neural crest formation, FGF and BMP, could mimic vegetal inducing signals. Treatment of b4.2 explants with either bFGF (100ng/ml) or BMP4 (300ng/ml) proteins from the 16-cell stage led to formation of acetylated α-tubulin-positive ESNs at the larval stage (bFGF 100%, *n* = 26; BMP4 81%, *n* = 47; Figure 3C and 3D). The formation of ESNs was accompanied by the activation of the midline marker *KLF1/2/4* at the early tailbud stage (bFGF 94%, *n* = 18; BMP4 88%, *n* = 26; Figure 3G and 3H). In both cases, the expression of the lateral marker *citb003j16* was suppressed (bFGF 4% of positive explants, *n* = 27; BMP4 6%, *n* = 16; Figure 3K and 3L). Interestingly, *KLF1/2/4* was expressed throughout the BMP4-treated explants, but only in some cells of the bFGF-treated ones. We asked whether *KLF1/2/4*-negative cells in bFGF-treated explants could be of neural character, as FGF is involved in inducing central nervous system (CNS) identity in the anterior a-line ectoderm [ 27, 35]. Indeed, expression of the tail nerve cord marker *cilv038e16* [ 29] was activated by bFGF but not by BMP4 in b4.2 explants at early tailbud stages (BSA 10%, *n* = 20; bFGF 90%, *n* = 20; BMP4 4%, *n* = 27; Figure 3M– 3P), showing that bFGF-treated explants comprise CNS and epidermis midline cells.

**Figure 4:**
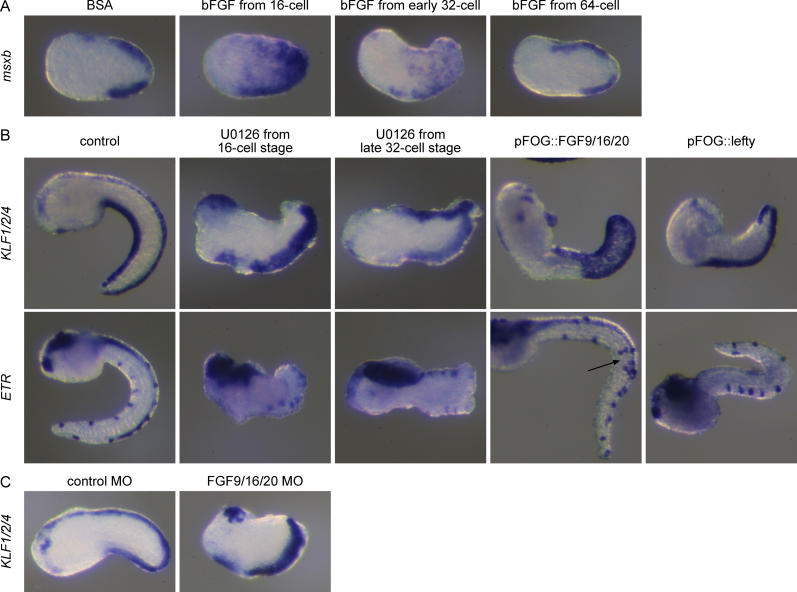
The FGF and Nodal Pathways Control Dorsal Midline and CESN Formation (A) bFGF-treated embryos show ectopic expression of the midline marker *msxb* at the late neurula stage when the treatment starts at the 16-cell or early 32-cell stages. Embryos do not respond to bFGF at the 64-cell stage. (B) Blocking Erk activity with the pharmacological inhibitor U0126 from the 16-cell stage abolishes dorsal *KLF1/2/4* and *ETR* expression at tailbud stages, while treatment from the late 32-cell stage has no effect on these markers. FGF9/16/20 is sufficient to induce ectopic midline and ESNs formation (arrow), while Lefty prevents dorsal midline formation. (C) FGF9/16/20 MO–injected embryos show a loss of dorsal *KLF1/2/4* expression while embryos injected with a control MO are not affected. (Lateral view, anterior to the left, dorsal to the top in all panels).

**Figure 5:**
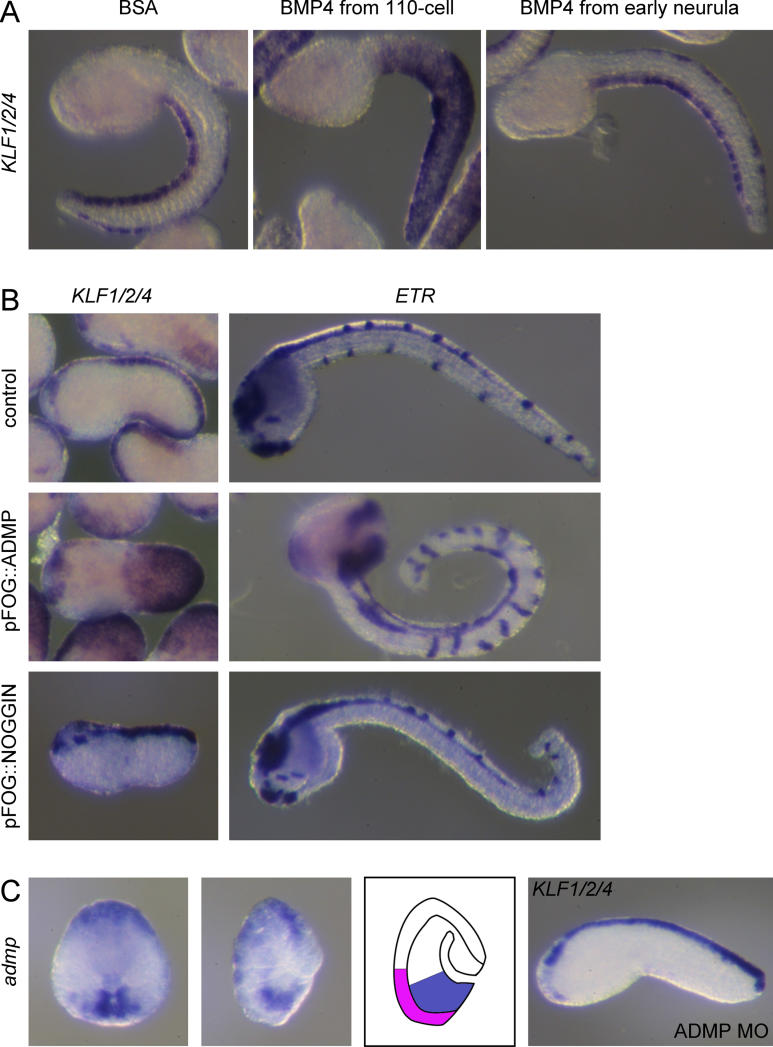
The BMP Pathway Controls Ventral Midline and CESNs Formation (A) BMP4-treated embryos show ectopic expression of the midline marker *KLF1/2/4* at the tailbud stage when the treatment starts at the 110-cell stage. Embryos do not respond to BMP4 at the early neurula stage. (B) ADMP overexpression transforms the entire tail epidermis into *KLF1/2/4-*positive tissue containing *ETR*-positive neurons. NOGGIN overexpression abolishes ventral midline formation. (C) The BMP ligand ADMP is expressed in B-line midline vegetal cells (blue on scheme) underlying the ventral midline precursors (pink on scheme) at the mid-gastrula stage. ADMP MO–injected embryos do not express *KLF1/2/4* ventrally. (Lateral view, anterior to the left, dorsal to the top in all panels except in C for *admp* expression pattern: anterior to the top, vegetal view (first image), sagittal section (second image and scheme)).

To determine when animal cells are competent to respond to FGF and BMP in vivo, we treated whole embryos with the recombinant proteins at different stages. bFGF-treated embryos develop with an aberrant morphology, making the analysis of late markers, such as *KLF1/2/4,* difficult to interpret (unpublished data) *.* We therefore analyzed the expression of *msxb,* another midline marker, at the late neurula stage before morphological defects complicate the analysis. Ectopic *msxb* expression was induced by bFGF when the treatment started between the eight-cell and early 32-cell stages, but not at the 64-cell stage ( Figure 4). By contrast, treatments from both the eight-cell stage and the 110-cell stage with BMP4 protein led to a robust ectopic expression of *KLF1/2/4* in the entire tail epidermis at the mid-tailbud stages with minor effects on the overall morphology ( Figure 5). In these embryos, *β-thymosin-like-* and acetylated α-tubulin-positive ESNs are found scattered on the entire tail epidermis (unpublished data). BMP4 treatment from the early neurula stage had no effect.

In summary, both FGF and BMP pathways activation induce midline fate and CESN differentiation in the tail epidermis but with a different time window.

### The Formation of the Dorsal Midline Is Dependent upon an Early FGF9/16/20-Erk-Nodal Signalling Cascade

We have shown that bFGF can induce tail nerve cord and tail epidermis midline fates in isolated b4.2 blastomeres. Our lineage analysis indicates that the dorsal midline shares lineage history with the tail dorsal nerve cord: the two tissues derive from the b6.5 blastomere at the 32-cell stage (with the exception of the very posterior dorsal midline, derived from b8.21). The two fates separate at the 110-cell stage [ 33]. The Erk pathway is activated in the b6.5 blastomere at the 32-cell stage by the FGF9/16/20 signal emanating from underlying vegetal cells [ 27, 35], suggesting that FGF might be involved in dorsal midline formation.

We first tested whether the Erk pathway controls midline fate in b6.5. Embryos treated with the mitogen-activated/extracellular signal-regulated protein kinase (MEK) pharmacological inhibitor U0126 from the 16-cell stage showed a loss of expression of *KLF1/2/4, ETR,* and *Hesb* dorsally, but not ventrally ( Figure 4B and unpublished data). By contrast, embryos treated with U0126 from the late 32-cell stage expressed these genes both dorsally and ventrally. We next tested the ability of FGF9/16/20 to behave as a midline inducer by overexpressing it in the entire ectoderm using the ectodermal pFOG enhancer (see the section Materials and Methods). Electroporated embryos showed ectopic expression of midline markers *(KLF1/2/4* and *ETR)* and repression of the lateral marker *Emx* ( Figure 4B and unpublished data). The time window for Erk dependency and the effects of *fgf9/16/20* overexpression make FGF9/16/20 a good candidate inducer. Indeed, inactivation of *fgf9/16/20* mRNA by injection of an antisense morpholino oligonucleotide (MO) led to a loss of *KLF1/2/4* expression in the dorsal, but not in the ventral midline (control MO: 100% strong dorsal expression, *n* = 41; *fgf9/16/20* MO: 9% weak dorsal expression, *n* = 23; Figure 4C). In conclusion, the FGF/Erk pathway is necessary and sufficient before the late 32-cell stage to define the dorsal region of the tail epidermis.

Hudson and Yasuo have shown that *fgf9/16/20* activates *Nodal* expression in the b6.5 blastomeres at the 32-cell stage and that *Nodal* acts downstream of *fgf9/16/20* to pattern the CNS including the b6.5 derivatives [ 36]. We find that overexpression of the secreted inhibitor of Nodal, *Lefty* [ 37, 38], suppressed expression of *KLF1/2/4* and *ETR* in the dorsal midline with the exception of the very posterior portion, the ventral midline being unaffected ( Figure 4B). *Nodal* is not sufficient to drive ectopic midline fate when overexpressed (unpublished data), suggesting that additional factors act alongside Nodal downstream of *fgf9/16/20*.

In summary, *fgf9/16/20* signalling, via the Erk pathway, and Nodal signalling are required to specify the dorsal epidermis midline, the ventral midline being independently specified.

### Ventral Midline Formation Requires BMP Signalling

BMP could be the signal required to specify the ventral midline. We first checked whether endogenous BMP ligands could reproduce the phenotype triggered by human BMP4 protein treatment. Overexpression of the ligands BMP2/4 or ADMP, using electroporation with the pFOG driver, suppressed lateral identity (loss of *Emx* expression; unpublished data) and induced midline identity and ESN formation ( Figure 5B).

We then tested whether the BMP pathway is required in vivo. Overexpression of the secreted inhibitor Noggin [ 39, 40] by either injection of Xenopus laevis Noggin mRNA or electroporation of pFOG::Ci-noggin led to a specific loss of *KLF1/2/4* and *ETR* expressions in the ventral midline without affecting dorsal expression ( Figure 5B and unpublished data). Marker analyses indicate that the ventral midline and the ventral medio-lateral domains were transformed into the lateral domain (unpublished data). Further supporting the role of BMP signalling, injection of a morpholino targeting the BMP specific Smad transducer Smad1/5 led to the absence of ventral midline formation (unpublished data). The BMP pathway is thus necessary and sufficient to define the ventral midline identity of the tail epidermis.

Seven BMP ligands have been identified in the C. intestinalis genome [ 41]. Three of them *(bmp2/4, bmp5/7-like,* and *admp)* are expressed in the B-line posterior vegetal cells underlying the future ventral epidermis midline ( Figure 5C and unpublished data). *admp* starts to be expressed there before gastrulation, when animal cells are competent to respond to exogenous BMP4 protein. By contrast, *bmp2/4* and *bmp5/7-like* expression starts during gastrulation. We thus tested the requirement of *admp* by morpholino-mediated knock-down. Injected embryos developed with no major morphological defect but completely lacked *KLF1/2/4* ventral expression (control MO: 76% strong ventral expression, *n* = 41; *admp* MO: 0% ventral expression, *n* = 39; Figure 5C). This phenotype could be rescued by co-injecting the pFOG::ADMP construct whose mRNA product is not targeted by the morpholino (unpublished data). Thus, both gain- and loss-of-function experiments demonstrate that *admp* expressed in the B-line medial vegetal cells acts as an endogenous inducer of the ventral epidermis midline.

### Delta/Notch Signalling Controls the Number of CESNs

**Figure 6:**
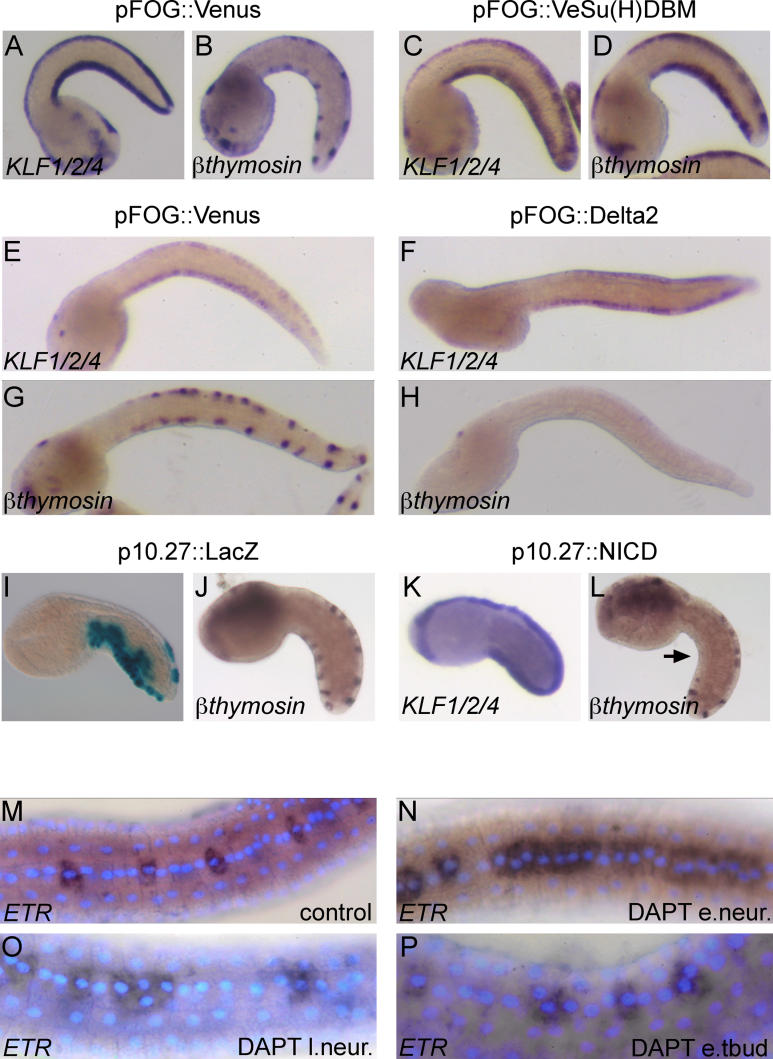
Delta/Notch Signalling Controls the Number of CESNs within Neurogenic Midlines (A,B,E,G) Electroporation of the construct pFOG::Venus (a bright YFP) has no effect on the midline domain and the CESNs, respectively, revealed by the *KLF1/2/4* and *β-thymosin-like* probes. The expression levels of *KLF1/2/4* at mid- to late tailbud stages (E) are lower than at mid-tailbud stages (A). (C and D) Electroporation of the dominant-negative construct pFOG::Venus-Su(H)DBM does not affect the expression of *KLF1/2/4* but leads to an increase in the number of *β-thymosin-like-*positive cells. (F and H) Electroporation of pFOG::Delta2 results in a loss of *β-thymosin-like-*positive cells, without affecting *KLF1/2/4* expression. (I and J) The construct p10.27::LacZ preferentially drives expression of β-galactosidase in the ventral tail epidermis (I), without affecting the development of CESNs (J). (K and L) Electroporation of p10.27::NICD, carrying an active form of Notch, does not affect the formation of the midline domain (K), but leads to a loss of CESNs in the ventral midline (arrow in L). (M–P) Treatment with the γ-secretase inhibitor, DAPT, at various developmental stages results in varying degrees of midline epidermal cell replacement by *ETR*-positive CESN precursors. e.neur., early neurula; l.neur., late neurula; and e.tbud, early tailbud.

Our results show that the midlines are neurogenic regions with the competence to generate both epidermal cells and CESNs. Given that *Delta2,* a putative DSL-type ligand for Notch, is expressed in scattered midline cells before the last division of the CESN lineage ( Figure S2), we explored the possibility that Notch signalling regulates the number of CESNs. We electroporated fertilized eggs with dominant-negative or constitutively active forms of Notch pathway molecules under the control of the pFOG driver ( Figure 6). Electroporation of the construct pFOG::Venus-Su(H)DBM, carrying a fusion between the bright form of YFP, Venus [ 42], and a dominant-blocking form of the Notch nuclear effector, Suppressor of Hairless Su(H) [ 43], resulted in a robust fluorescence in all the epidermal cell nuclei at mid- to late tailbud stage (unpublished data). The overall embryo morphology and the size of the medio-lateral epidermal domains were not affected, but the CESN marker *β-thymosin-like* was expressed throughout the medial domain, rather than in isolated CESN pairs as is the case for the control, pFOG::Venus-electroporated embryos ( Figure 6A– 6D). *β-thymosin-like-* positive cells were never observed in the medio-lateral or lateral domains.

To address the effect of Notch pathway activation on the formation of CESNs, we adopted a two-pronged strategy: 1) we electroporated embryos with a construct carrying the putative Notch ligand *Delta2* under the control of the pFOG promoter. In these embryos, the formation of the tail was not affected, but *β-thymosin-like-*positive cells were lost from the tail epidermis ( Figure 6F and 6H); 2) we took advantage of the driver p10.27 [ 44] that controls expression in the tail epidermis from the neurula stage. X-Gal staining of embryos electroporated with the construct p10.27::LacZ showed preferential expression of the transgene in the ventral tail epidermis ( Figure 6I). In embryos electroporated with a plasmid carrying the constitutively active Notch intracellular domain (NICD) under the control of p10.27, *KLF1/2/4* expression was unaffected, but *β-thymosin-like-*positive cells were absent or reduced in the ventral tail midline ( Figure 6K and 6L). Thus, activation and block of the Notch pathway, respectively, result in a decrease and in an increase in the numbers of CESNs.

### Temporal Requirement of Notch Pathway in CESN Generation

Regulated intramembrane proteolysis of Notch by γ-secretase is required to generate the active form NICD and is therefore a crucial step in Notch signalling [ 45]. To gain a better insight into the temporal requirement of the Delta/Notch pathway and its mechanisms of action in the determination of CESN numbers, embryos at early neurula, late neurula, and early tailbud stage were treated with the γ-secretase inhibitor, DAPT [ 46], followed by *ETR* in situ hybridization and DAPI staining at the late tailbud stage, prior to the final cell divisions that generate CESN pairs.

As shown in Figure 6 (M–P), the severity of the phenotype changes according to the time of DAPT treatment. In embryos incubated with DAPT from the early neurula stage, most of the medial domain was constituted by *ETR-*positive cells and only few epidermal cells were present ( Figure 6N); in embryos treated from the late neurula stage, islands of 2–3 *ETR-*positive cells alternated with regions of epidermal cells ( Figure 6O); embryos treated at the early tailbud stage showed no significant change in the number and distribution of ETR-positive cells compared with control embryos ( Figure 6M and 6P). In all cases, the expression of *KLF1/2/4* in the medial domain appeared normal (unpublished data); medio-lateral and lateral domains did not appear to be affected and never contained *ETR-*positive cells.

Thus, the competence of the midline domain to generate supernumerary CESNs when the Notch pathway is blocked progressively declines during development. Moreover, the inverse relationship between the number of CESNs and epidermal cells in the midline domains strongly suggests the existence of a Notch-mediated lateral-inhibition phenomenon responsible for preventing excess midline cells from adopting a CESN fate.

## Discussion

**Figure 7:**
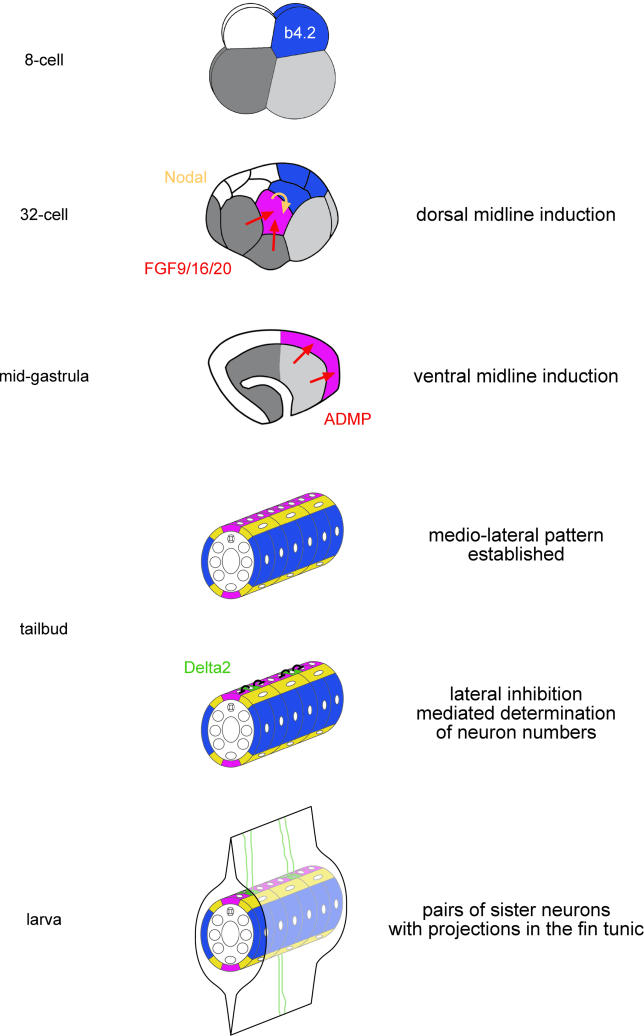
A Model for CESN Formation The tail epidermis derives from the posterior animal b4.2 blastomeres. At the 32-cell stage, FGF9/16/20 induces *Nodal* and dorsal midline identity in the b6.5 blastomere. At the mid-gastrula stages, ADMP induces ventral midline fate in the overlying epidermis. These first inductive processes lead to a medio-laterally patterned epidermis comprising eight rows of cells in cross section. Finally, Notch signalling controls the number of CESNs in the midline through lateral inhibition.

Our results allow us to build a detailed scenario for CESN specification from cleavage stages to larval stages ( Figure 7). The entire tail epidermis derives from the b4.2 blastomere at the eight-cell stage, which is originally endowed with a lateral identity. In a first step, during cleavage and gastrulation stages, the midlines are induced as neurogenic regions by vegetal signals: FGF9/16/20, partially relayed by Nodal, induces the dorsal midline, and ADMP the ventral one. All b-line cells being competent to respond to these signals, the restriction of midline fate to a subset of precursors likely originates from the localized expression of the inducers. In a second step, from neurula to early tailbud stages, the progenitors of the CESNs are singled out within the neurogenic midlines and their number is controlled through a process of Notch-mediated lateral inhibition likely triggered by Delta2. Finally, each selected progenitor undergoes a last cell division to generate a pair of CESNs which differentiate at the larval stages and send projections into the fin tunic.

Here, we discuss several issues concerning the mechanisms of medio-lateral patterning, the role of the Notch pathway, and the physiological function of the CESNs. Finally, by comparing our results with other systems, we uncover a link between median fin and PNS formation which could provide a new and original perspective on the evolutionary origin of these tissues.

### Patterning of the Tail Epidermis

#### Dorso–ventral patterning.

At tailbud stages, the ventral and dorsal midlines appear identical based on morphology and gene expression. Yet, we have demonstrated that two separate pathways are required for their specification. It is possible that FGF9/16/20 and ADMP directly activate pan-midline genes such as *KLF1/2/4*. Alternatively, these molecules might act indirectly through ventral and dorsal intermediates that would in turn activate pan-midline markers. In agreement with this proposition, the epidermis is patterned along its dorso–ventral axis (dorsal markers *chordin* and *Pax3/7* and ventral markers *bmp2/4, Smad6/7,* and *Tbx2/3* [ 30, 36, 47] and our unpublished observations). Testing these hypotheses will require understanding the epistatic relationships and deciphering cis-regulatory elements controlling epidermis marker expression, two approaches that the *Ciona* system is well suited for.

#### Medio-lateral domain specification.

We have shown that, in addition to the midlines, BMP and FGF signalling are also required for the formation of the ventral and dorsal medio-lateral domains, respectively. It is yet unclear whether these pathways directly specify the medio-lateral fate. They might induce midline and medio-lateral fates in a dose-dependent manner. Another possibility is that midline fate specification might be a prerequisite for medio-lateral fate to occur. In this latter case, two non–mutually exclusive scenarios can be proposed. In a relay model, the medial row would act as a signalling zone that laterally induces the medio-lateral fate. Alternatively, the juxtaposition of the midline and lateral domains could lead to the formation of a third, medio-lateral fate.

### The Role of Notch Signalling

We showed that the tail epidermis midline is a neurogenic region within which all cells transiently express a homologue of the proneural *Achaete-Scute* genes, *Ci-Achaete-Scute a-like2* ( Figure S2). The Notch pathway negatively controls the number of midline cells that differentiate as CESN. However, its exact role remains to be elucidated. A Notch-dependent process of lateral inhibition is at work to prevent an excessive number of midline cells from adopting a CESN fate. It is less clear whether Delta/Notch signalling also drives the initial step of CESN precursor selection via lateral competition among midline cells. Expression of Notch ligand *Delta2* never appears throughout the entire midline. Instead, it is progressively turned on in scattered cells following a caudo-rostral wave ( Figure S2). This pattern resembles what was observed in vertebrates, where *Delta1* is expressed by nascent neurons [ 48], rather than in *Drosophila,* where *Delta* is expressed throughout the proneural clusters [ 49]. It is likely that the CESN precursors are initially chosen within the neurogenic midline by Delta2-independent factors that activate *Delta2* expression. This in turn would relay an inhibitory signal to adjacent cells so that they do not embark on a CESN program but rather develop as epidermal cells. Given the morphological simplicity of the midline epidermis and the fact that only one cell fate choice is at play (CESNs versus epidermal cells), this model could be exploited to study at the single-cell level the mechanisms linking neural induction and neurogenesis in chordates, in particular the transcriptional regulation of neurogenic gene activity.

### Function of the CESNs

Based on their morphology, CESNs have been proposed to be mechanoreceptors controlling swimming behavior [ 23]. Strong evidence for this function is lacking, however, but several approaches, spurned by recent molecular developments, could now be used to readdress this issue. First, transient receptor potential channels are key actors of sensations including mechanosensation [ 50, 51]. Determining which of these genes are specifically expressed in the CESNs should significantly increase our understanding of their function. This will be feasible since the 27 members of this large family present in the *Ciona* genome have been precisely annotated [ 52].

Likewise, the availability of a collection of cDNAs for neurotransmitter biosynthetic enzymes makes it possible to determine the neurotransmitter phenotype of these neurons. Finally, the function of a neuron is determined by its projections. *Ciona* CESNs send neurites towards the trunk CNS, but their targets are unknown [ 22, 23]. Determining the targets of their projections, by using for example *β-thymosin-like* regulatory sequences to drive a reporter such as wheat germ agglutinin [ 53], should answer this issue.

Assuming the CESNs are indeed mechanosensory, they are most likely involved either in the detection of water vibrations, triggering escape response, or in the actual control of swimming behavior, which can be studied in detail (reviewed in [ 54]). The physiological role of the ascidian sensory neurons could be directly addressed by the specific suppression of their formation, through the overexpression of Delta2 in part or the whole tail epidermis for instance. These experiments could additionally indicate whether ventral and dorsal CESNs assume identical functions.

### The Dorsal Midline: A Homologue of the Vertebrate Neural Plate Border?

The vertebrate PNS originates from neurogenic placodes and neural crest which are derived from the neural plate border. The *Ciona* dorsal midline epidermis shares with the vertebrate neural plate border its position within the embryo and the expression of orthologous genes [ 3, 18– 21]. Our study has revealed key aspects of its specification. We have shown that, as the vertebrate neural plate border, it is induced by the underlying mesendoderm and that FGF signalling participates in this induction. While the FGF pathway is widely exploited during embryogenesis and, on its own, brings poor evolutionary information, our study strengthens the similarity between ascidian dorsal midline epidermis and vertebrate neural plate border specification mechanisms. Moreover, the FGF pathway appears to have a conserved function in both PNS and CNS induction in chordates [ 55]. However, we have also uncovered important differences with neural plate border induction: the BMP pathway is not involved, while the Nodal pathway is. In conclusion, the common ancestor of urochordates and vertebrates may possess a neural plate border–like structure specified by induction through FGF pathway.

Nevertheless, the *Ciona* dorsal midline epidermis is not equivalent to the vertebrate neural plate border derivatives: the neural crest or placodes. It does not express homologues of markers for these structures [ 3, 13, 18– 21]. Moreover, our cell lineage analysis clearly demonstrates that two defining traits of neural crest and placodes (migratory ability and multipotency) could not be detected in ascidian embryos. How the ancestral neural plate border–like structure gave rise to CESNs in ascidians and to neural crest and placodes in vertebrates may be envisaged through a number of scenarios. To build plausible hypotheses, the identification of a vertebrate counterpart for the CESN might be crucial. According to morphology, function, or location along the body axis, few candidates derived from the neural plate border can be proposed, albeit none meet all criteria. The placodal derivatives include otic and lateral line mechanosensors and olfactory neurons. While the first are secondary (axon-less) neurons, the later are primary neurons arising within the olfactory epithelium but are only present in the cephalic region and their function is different from the one commonly attributed to CESNs. Finally, the caudally located Rohon-Beard neurons are primary mechanoreceptors that innervate the skin, project rostrally towards the brain, and control early swimming behavior, but are located within the spinal cord. A better knowledge of CESN function, physiology, and gene expression profile should enable appropriate comparisons.

### Epidermis Patterning and Fin Formation

While we initially analyzed epidermal patterning to understand CESN formation, we would like to propose that this patterning could also play a major role in fin formation. The *Ciona* larval fin is a specialization of the tunic, the acellular matrix that surrounds the larva and that unifies the urochordates (also known as tunicates). While the tunic is rather thin around the trunk and the flanks of the tail, it is massively expanded along the dorsal and ventral tail midlines to form a large median fin with essential hydrodynamic properties for swimming [ 54]. The tunic is a cellulose-based extracellular matrix secreted by the epidermal cells [ 56, 57], but very little is know about the genetic and biochemical pathways that control its secretion. To our knowledge, the only gene expressed in the epidermis and having a function in tunic synthesis is the gene coding for cellulose synthase [ 58– 60]. Increased production of tunic material or chemical modifications at the level of the midlines are likely causes for fin formation. It would thus be interesting to look for genes involved in polysaccharides synthesis or modifications more specifically expressed in the midlines, as they may constitute the link between patterning genes and the physiological machinery at work in the animal. Interestingly, preliminary results indicate that ectopic midline formation or loss of ventral midline, by manipulating the BMP pathway, leads to ectopic fin formation or loss of ventral fin, respectively (SD, unpublished data).

With respect to its function and position along the body axis, the ascidian acellular fin is analogous to the median fin of lower vertebrates, an ancestral structure already present in extinct primitive vertebrates [ 9, 61– 63]. Interestingly, the *Ciona* tail epidermis midlines, which are likely involved in fin formation, express genes such as *msxb* and *Dll-C,* whose homologues are expressed in zebrafish median fin [ 64, 65]. In frog, ventral and dorsal fins originate from separate embryonic regions and are independently induced [ 66]. The dorsal fin arises from dorsal epidermis lateral to the neural crest and is induced by this tissue, while the ventral fin arises from ventral medial epidermis close to the blastopore and is induced by underlying ventral mesoderm. Zebrafish mutants and conditional inactivation experiments demonstrate a requirement for the BMP pathway in ventral fin specification [ 67– 69]. These observations reveal striking similarities to midline formation in *Ciona*: both fate maps and inducing mechanisms appear to be conserved ( Figure S3). Our study thus provides a scenario for median fin origin in chordates.

### Neurogenesis and Fin Formation at the Midlines: An Evolutionary Perspective

The ascidian dorsal and ventral midlines are the regions where ESNs and fin form. In the present section, we would like to propose that this coupling between PNS and fin formation at the midlines might be important to understand the evolution of PNS formation in chordates. We shall first consider whether the common ancestor of all chordates possessed ventral and dorsal midlines endowed with both neurogenic and fin-forming potential. Chordates comprise cephalochordates (amphioxus), urochordates, and vertebrates, with urochordates being the closest relatives of vertebrates [ 10, 11]. A median fin is observed in lower vertebrates, amphioxus, and urochordates [ 56, 62, 63, 70], and thus probably represents an ancestral feature of the chordate phylum. While the median fin is clearly associated with PNS in ascidians, the current data on amphioxus cannot give a definitive answer. Amphioxus possess ESNs that likely originate from the epidermis, similarly to what we described for *Ciona* [ 71– 76]. Neurogenesis in the epidermis would thus constitute a primitive feature among chordates. While ESNs can be found at various dorso–ventral positions, it is not known whether they are born at the same place where they differentiate or whether they come from the midlines like in *Ciona*. Interestingly, ventro-lateral ESNs apparently originate from the ventral epidermis midline before migrating to their final position [ 75]. The ancestor of chordates might thus have possessed the association of a median fin with both a dorsal and a ventral neurogenic midline. By contrast, this association is not present in vertebrates. While the ventral and dorsal fins have been kept, the ventral midline epidermis has lost its neurogenic potential, and a migratory neural crest has emerged on the dorsal side.

The proposed scenario has additional implications concerning the molecular control of median fin and PNS formation. It implies that the genetic networks controlling median fin and PNS formation in vertebrates should partially overlap. When considering factors acting upstream of these networks, we would like to propose two alternate scenarios. The ancestor may have used the FGF and BMP pathways together similarly in both dorsal and ventral midlines. A simplification in ascidians led to a separate usage between the two midlines, while the two pathways have been kept dorsally for neural plate border formation in vertebrates. Alternatively, the dichotomic use of FGF and BMP we uncovered in *Ciona* was already present ancestrally. The ventral BMP program may have been recruited dorsally in the vertebrate lineage for neural plate border induction. The best way to test the above speculative scenarios would be to get a better understanding of fin and PNS formation in amphioxus, where functional analysis of developmental processes has made significant progress in recent years [ 77, 78].

### Conclusion: Towards a Complete Gene Network

Our study has implications for both molecular development and evo–devo fields. We have presented a new system that enables understanding cell fate decision at the cell level in a spatially and temporally controlled manner. We believe this provides a scaffold upon which a network taking into account individual cell history and location as well as genetic regulation may be built. Importantly, *Ciona* is a well-suited organism for this task [ 79]. A gene regulatory network should first enable us to represent the diversity of the factors involved, and point both to convergent subnetworks (e.g., integration of the dorso–ventral patterning into pan-midline program) and to divergent outputs (e.g., neurogenesis versus fin formation). An even more appealing outcome of building a network is phylogenetic comparison. If the network governing *Ciona* midline formation is comparable to the vertebrate neural plate border network, we might be able to pinpoint important evolutionary changes or decipher mechanisms of gene cooption paving the way to the emergence of true neural crest and placodes. Moreover, this might be a way to test the proposed link between median fin and vertebrate PNS. As we and others such as Holland [ 76] understand the difficulty of determining homologies, we think the more the network is detailed, the more we will be able to understand evolution and build working hypotheses, some of which could be functionally tested.

## Materials and Methods

### C. intestinalis embryos.

Embryo culture, blastomere isolation, U0126 and protein treatments, and DiI labelling were as described [ 26, 27]. Recombinant human BMP4 protein was purchased from R&D Systems (Minneapolis, Minnesota, United States). The γ-secretase inhibitor DAPT, also known as γ-secretase inhibitor IX (Calbiochem, San Diego, California, United States), was prepared as a 20mM stock solution in DMSO. Because DAPT is poorly soluble in seawater, the precise working concentration could not be determined. The most consistent results were obtained by diluting the stock solution 200 times in seawater, followed by filtering to remove DAPT precipitate immediately prior to embryo treatment.

### Whole mount in situ hybridization and immunochemistry.

Whole mount in situ hybridization and antibody staining were performed according to [ 26]. Acetylated α-tubulin and β-tubulin staining were performed with the 6–11B-1 and 2-28-33 monoclonal antibodies (Sigma-Aldrich, St. Louis, Missouri, United States). Confocal images were acquired using a Zeiss LSM510 inverted microscope. 3-D stacks were processed using the Zeiss embedded software and Imaris (Bitplane, Zurich, Switzerland).

### Generation and electroporation of expression vectors.

Overexpression was performed using transient transgenesis by electroporation as described in [ 35], but all volumes were scaled down to a half. 30 μg to 100 μg of plasmid DNA were used. Most plasmids were generated using the Gateway system (Invitrogen, Carlsbad, California, United States). Detailed procedures and destination vectors used will be described elsewhere (Roure et al., in preparation). Entry clones were generated by PCR using Pfx polymerase (Promega, Madison, Wisconsin, United States) and designed to contain the entire ORF of interest preceded by a Kozak sequence or an N-terminal YFP Venus tag [ 42]. The list of template cDNAs and primers used can be found in Table S3. The only construct containing UTRs is *Delta2* whose entry clone directly comes from a Gateway compatible library (ciem846i12, a gift from Y. Satou). The plasmid pRN3 Ci-Su(H)DBM, used as a template to generate the fusion construct pFOG::Venus-Su(H)DBM, was a gift from H. Yasuo and was based on the citb043k14 clone. The two destination vectors, pFOG::Rfa and pFOG::Venus-Rfa, contain 2kb upstream of the *friend-of- GATA (FOG)* gene, driving pan-ectodermal expression from the 16-cell stage (Rothbächer et al., in preparation). The p10.27::NICD vector was generated by PCR-amplifying zebrafish NICD (Notch1a intracellular domain) from the pCS2+NICD vector [ 80] and subcloning it into the KpnI and EcoRI sites of the p10.27CES plasmid [ 44], in lieu of the LacZ ORF. The 10::27 sequence corresponds to 1.5 kb of *Ciona* genomic DNA that drive expression in the tail epidermis from neurula stages ([ 44]; and our own observation). Despite mosaic inheritance of electroporated DNA, the electroporation conditions we used yield similar phenotypes in more than 80% of the embryos. Each marker was typically analyzed on 30–50 embryos per experiment, each repeated at least twice.

### mRNA and morpholinos microinjection.

mRNA synthesis and injections were performed as in [ 35]. Synthetic mRNAs for Xenopus laevis noggin and histone2B-eYFP were generated using the pBSRN3XlnogginΔ5′ construct [ 81] and pCSH2BEYFP construct [ 82], and injected at 1.0 μg/μl. The MOs (Gene Tools, Philomath, Oregon, United States) against *fgf9/16/20* (FGF9-MO [ 35]), *admp* (ADMP-MO: ATCTGTTTCCTGAGATGTCAAACTC, designed in the 5′UTR) and *Smad1/5* (SMAD15-MO: AACCAAATAAACTTGTCATCGCCAT, designed around the ATG) were injected at 0.25–0.50 mM. The effect of ADMP-MO could be rescued by pFOG::ADMP, which does not include the 5′UTR sequence.

## Supporting Information

Figure S1 Contribution of Each 110-Cell Stage b-Line Blastomere to the Larval Tail Epidermis (2.0 MB TIF)

Figure S2 Different Expression Patterns of the *Ci-achaete-scute-2a* and *Ci-delta2* Genes in the Neurogenic Ventral Tail Midline Region
*Ci-achaete-scute-2a* is expressed throughout the prospective midline region from neurula to mid-tailbud stage and is subsequently rapidly downregulated. The expression of *Ci-delta2* is first initiated in scattered cells of the posterior prospective midline (red arrows), progressively propagates to isolated cells of the anterior midline, and persists through tailbud stages in the CESNs. Itb, initial tailbud; etb, early tailbud; emtb, early to mid-tailbud; mtb, mid-tailbud. The ventral midline domain can be first identified as a roughly triangular cell field on the ventral posterior aspect of the neurula stage embryo. Tail elongation, cell intercalation, and cell shape changes progressively convert it into a single cell–wide row stretching along the ventral aspect of the tail.
(5.6 MB TIF)

Figure S3 Comparison between C. intestinalis Midlines Fate Map and Xenopus laevis Median Fin Fate Map
The fate map for *Ciona* is based on our lineage results. The *Xenopus* fate map is adapted from [ 66].
(2.4 MB TIF)

Table S1 Medio-Lateral and CESNs Markers (48 KB DOC)

Table S2 Qualitative and Quantitative Contribution to the Tail Epidermis of b-Line Blastomeres at the 110-Cell Stage (4 KB PDF)

Table S3 List of Primers Used in the Present Study (53 KB DOC)

## Abbreviations

BMP: bone morphogenetic protein
CESN: caudal epidermal sensory neuron
CNS: central nervous system
Erk: extracellular signal-regulated kinase
ESN: epidermal sensory neuron
FGF: fibroblast growth factor
*FOG*: *friend-of- GATA *
pFOG: *FOG* promoter
MEK: mitogen-activated or extracellular signal-regulated protein kinase
MO: antisense morpholino oligonucleotide
NICD: Notch intracellular domain
PNS: peripheral nervous system
Su(H): Suppressor-of-Hairless
