# Supplementary material for: Formation of the Ascidian Epidermal Sensory Neurons: Insights into the Origin of the Chordate Peripheral Nervous System
Source: PLoS Biol. 2006 Jun 27;4(7):e225. doi: 10.1371/journal.pbio.0040225 (PMC1481523; doi:10.1371/journal.pbio.0040225)
Supplement: Supplementary file 1 [file pbio.0040225.st002.pdf]

Table S2. Qualitative and quantitative contribution to the tail epidermis of b-line blastomeres at the 110-cell stage.

DiI-labelled blastomere	n. of DiI+cells at late tb stage	n. of CEN pairs	n. of epidermal cells					Σ=n. of epidermal cells + n. of CEN pairs
			dm	dml	l	vml	vm	
b8.22 (n=4)	16	0		8	8			16
b8.23 (n=15)	16	0		8	8			16
b8.24 (n=25)	16	0		16*				16
b8.25 (n=17)	16	0			8	8		16
b8.26 (n=3)	16	0			16			16
b8.29 (n=6)	16	0			16			16
b8.30 (n=5)	16	0			16*			16
b8.18 (n=10)	15-21	1-5	8-15					14 (n=6)<br>16 (n=4)
b8.20 (n=11)	16-17	0-1	15-16*					16
b8.21 (n=22)	12-13	0-1	3-4	8				12
b8.27 (n=30)	14-18	0-4#					10-14	14
b8.28 (n=8)	16-19	0-3				8	5-8	16
b8.31 (n=31)	16-19	0-3				8	5-8	16
b8.32 (n=18)	16-18	0-2				8*	5-8*	16
b9.62 (n=11)	8-10	0-2					6-8	8
b9.56 (n=5)	8-10	0-2					6-8	8

* A variable number of epidermal cells generated by the anteriormost b-line blastomeres, b8.24, b8.30, b8.20 and b8.32, contributes to the trunk epidermis

In addition to a variable number of CEN pairs, b8.27 also gives rise to a single ciliated cell at the tail tip
