# Supplementary material for: Formation of the Ascidian Epidermal Sensory Neurons: Insights into the Origin of the Chordate Peripheral Nervous System
Source: PLoS Biol. 2006 Jun 27;4(7):e225. doi: 10.1371/journal.pbio.0040225 (PMC1481523; doi:10.1371/journal.pbio.0040225)
Supplement: Supplementary file 2 [file pbio.0040225.st001.doc]

**Table S1.** Medio-lateral and CESNs markers.

| **Gene name** | **JGI gene ID** | **Clone used** | **Expression pattern in the tail epidermis** | | | |
| --- | --- | --- | --- | --- | --- | --- |
|  |  |  | **CESNs** | **Medial** | **Medio-lateral** | **Lateral** |
| *ETR* | ci0100147349 | citb028e11 | X |  |  |  |
| *POU-IV* | ci0100146655 | citb034g05 | X |  |  |  |
| *βthymosin-like* | ci0100151460 | cieg009o24 | X |  |  |  |
| *KLF1/2/4* | ci0100142734 | citb012d14 |  | X |  |  |
| *msxb* | ci0100152602 | cign067l18 |  | X |  |  |
| *Dll-C* | ci0100145240 | cilv080b23 |  | X |  |  |
| *Nkx-C* | ci0100150141  ci0100150167 | citb089b03 |  | X |  |  |
| *Achaete-Scute a-like2* | ci0100148570 | citb032n03 |  | X |  |  |
| *Hesb* | ci0100139157 | ciad039g19 |  |  | X |  |
| *Trypsin type protease 31* | ci0100142324 | citb003j16 |  |  | X | X |
| *Emx* | ci0100151065 | cilv052a23 |  |  |  | X |
| *FoxB* | ci0100145669 | cilv039e20 |  |  |  | X |
