# Supplementary material for: Formation of the Ascidian Epidermal Sensory Neurons: Insights into the Origin of the Chordate Peripheral Nervous System
Source: PLoS Biol. 2006 Jun 27;4(7):e225. doi: 10.1371/journal.pbio.0040225 (PMC1481523; doi:10.1371/journal.pbio.0040225)
Supplement: Supplementary file 3 [file pbio.0040225.st003.doc]

**Table S3**

| **Gene** | **Template clone** | **Primer** | **Kozak** |
| --- | --- | --- | --- |
| fgf9/16/20 | full length cDNA (Bertrand et al., 2003) | Fw: ggggacaagtttgtacaaaaaagcaggcTCCATATTGAAAATGTCTATGTTAAC  Rv: ggggaccactttgtacaagaaagctgggtCTCAGTAGAGTCGGCCAGAG | Yes |
| admp | cicl101j11 | Fw: ggggacaagtttgtacaaaaaagcaggcTCAGAAAAAATGTATCTTAGGCAGATAAC  Rv: ggggaccactttgtacaagaaagctgggtATTCATCTGCAGGCACACGATGC | Yes |
| bmp2/4 | cicl060n01 | Fw: aaaaagcaggcTCAGAAAAAATGGTGGCGCTTACGGATTGGA  Rv: agaaagctgggtCTATCTACACCCACAAGCTTGCACC | Yes |
| noggin | cilv027l15 | Fw: ggggacaagtttgtacaaaaaagcaggcTCAGAAAAAATGAATTTCGCTACTTGTTTTGC  Rv: ggggaccactttgtacaagaaagctgggtTCGAGTTAAGGGCATTGG | Yes |
| lefty | cign046m05 | Fw: ggggacaagtttgtacaaaaaagcaggcTCAATAATAATGTTTCCGATCAAG  Rv: ggggaccactttgtacaagaaagctgggtTTACACACCAAATACAC | Yes |
| nodal | cicl090l02 | Fw: ggggacaagtttgtacaaaaaagcaggcTCAGAAAAAATGATTTCTATGTTTAATATCGCTG  Rv: ggggaccactttgtacaagaaagctgggtATGTAAATTATCTGCAACCG | Yes |
| Su(H)DBM | pRN3 CiSu(H)DBM | Fw: ggggacaagtttgtacaaaaaagcaggcTTGTATCACCCCCACCACCT  Rv: ggggaccactttgtacaagaaagctgggtTCACGATGCAGTAGC | No |
| NICD | pCS2+NICD | Fw: CGGGGTACCGGATCCCATCGA  Rv: CCCCAATTGCTACTTGAAGGC | Yes |
